# Impaired Hand Grip Strength Correlates with Greater Disability and Symptom Severity in Post-COVID Myalgic Encephalomyelitis/Chronic Fatigue Syndrome

**DOI:** 10.3390/jcm13072153

**Published:** 2024-04-08

**Authors:** Anna Paffrath, Laura Kim, Claudia Kedor, Elisa Stein, Rebekka Rust, Helma Freitag, Uta Hoppmann, Leif G. Hanitsch, Judith Bellmann-Strobl, Kirsten Wittke, Carmen Scheibenbogen, Franziska Sotzny

**Affiliations:** 1 Institute of Medical Immunology, Charité—Universitätsmedizin Berlin, Corporate Member of Freie Universität Berlin and Humboldt-Universität zu Berlin, Augustenburger Platz 1, 13353 Berlin, Germany; anna.paffrath@charite.de (A.P.); claudia.kedor@charite.de (C.K.); elisa.stein@charite.de (E.S.); rebekka.rust@charite.de (R.R.); helma.freitag@gmx.de (H.F.); uta.hoppmann@charite.de (U.H.); leif-gunnar.hanitsch@charite.de (L.G.H.); kirsten.wittke@charite.de (K.W.); carmen.scheibenbogen@charite.de (C.S.); franziska.sotzny@charite.de (F.S.); 2 Experimental and Research Center (ECRC), Charité—Universitätsmedizin Berlin, Corporate Member of Freie Universität Berlin and Humboldt-Universität zu Berlin, Charitéplatz 1, 10117 Berlin, Germany; judith.bellmann-strobl@charite.de

**Keywords:** myalgic encephalomyelitis, post-COVID syndrome, chronic fatigue syndrome, hand grip strength, COVID-19

## Abstract

**Background:** Post-COVID syndrome (PCS) encompasses a diverse array of symptoms persisting beyond 3 months after acute SARS-CoV-2 infection, with mental as well as physical fatigue being the most frequent manifestations. **Methods:** In 144 female patients with PCS, hand grip strength (HGS) parameters were assessed as an objective measure of muscle fatigue, with 78 meeting the Canadian Consensus Criteria for postinfectious myalgic encephalomyelitis/chronic fatigue syndrome (ME/CFS). The severity of disability and key symptoms was evaluated using self-reported questionnaires. **Results:** Patients with ME/CFS exhibited heightened overall symptom severity, including lower physical function (*p* < 0.001), a greater degree of disability (*p* < 0.001), more severe fatigue (*p* < 0.001), postexertional malaise (*p* < 0.001), and autonomic dysfunction (*p* = 0.004) compared to other patients with PCS. While HGS was impaired similarly in all patients with PCS and exhibited a significant correlation with physical function across the entire patient group, HGS of patients with ME/CFS uniquely demonstrated associations with key symptoms. **Conclusions:** Thus, impaired HGS serves as an objective marker of physical function in patients with PCS. Only in patients meeting ME/CFS criteria is impaired HGS also associated with the severity of hallmark symptoms. This suggests a common mechanism for muscle fatigue and other symptoms in the ME/CFS subtype, distinct from that in other types of PCS.

## 1. Introduction

Large population-based studies have revealed that at least 3% of the population, mostly following mild infections, still suffer from numerous symptoms including fatigue and cognitive impairment following the resolution of the acute phase of the infection [1,2]. If symptoms occur within three months following a SARS-CoV-2 infection and persist for at least two months, the World Health Organization (WHO) classifies them as post-COVID condition or syndrome (PCS). These symptoms must affect everyday functioning and cannot be explained by an alternative diagnosis [3].

While some of these persistent symptoms may be caused by long-term inflammation and damage, there is little evidence for organ comorbidity in the majority of patients [4,5]. Typical symptoms include fatigue, exertional intolerance, postexertional malaise (PEM), cognitive and autonomic dysfunction, myalgia, and headaches [2]. In a subset of these patients, the diagnostic criteria for postinfectious myalgic encephalomyelitis/chronic fatigue syndrome (ME/CFS) are also met [6,7,8].

Postinfectious ME/CFS had affected approximately 800,000 to 2.5 million patients in the United States alone, even before the onset of the pandemic [9]. Given the high incidence of COVID-19, the prevalence of ME/CFS has likely experienced a substantial increase. This poses probably the most significant problem of the pandemic for healthcare and society, as ME/CFS not only profoundly affects patients’ quality of life but is also, in most cases, a chronic disease about which there is little knowledge and for which few are few resources available for disease management [9].

In our previous observational study, we found that patients diagnosed with post-COVID-ME/CFS generally did not show improvement in their health throughout the second year of the disease. In contrast, patients with non-ME/CFS PCS displayed a more favorable prognosis [8]. Most patients with PCS who experience fatigue but do not meet the diagnostic criteria for ME/CFS exhibit milder exertional intolerance with no or shorter PEM [8]. While there are clinical distinctions between PCS and ME/CFS, it is noteworthy that certain pathomechanisms have been recognized as potentially overlapping between the two conditions. The pathomechanisms identified in both PCS and ME/CFS include inflammation, autoantibodies, endothelial dysfunction leading to hypoperfusion, and impaired mitochondrial function [5,10].

The diagnosis of ME/CFS relies on specific sets of diagnostic criteria. Among these, the most commonly employed are the Canadian Consensus Criteria (CCC) and the Institute of Medicine (IOM) criteria. These criteria necessitate the presence of key symptoms, including fatigue, PEM, nonrefreshing sleep, cognitive impairment, and/or orthostatic intolerance [11]. However, it is crucial to note that the diagnosis of ME/CFS primarily relies on these clinical criteria, as there is currently no consensus on objective markers. This poses a challenge in making a diagnosis in clinical routine. Several studies have identified hand grip strength (HGS) as an objective marker of muscle fatigue in individuals with both ME/CFS [12,13,14] and PCS [8]. Assessment of HGS adds a valuable dimension to the diagnosis, offering a measurable parameter that complements the clinical criteria and enhances our ability to understand the physiological aspects of the condition.

Our study was designed with the primary objective of gaining a more comprehensive understanding of the symptom patterns observed in both PCS and ME/CFS. By examining HGS as an objective measure of muscle fatigue, our aim was to establish correlations between HGS and the levels of functional impairment and symptom severity assessed by questionnaires within distinct patient groups of PCS and ME/CFS.

## 2. Materials and Methods

### 2.1. Patients

A total of 144 female patients with persistent fatigue and other symptoms for at least 6 months following mild to moderate COVID-19 diagnosed with PCS according to the WHO criteria [3] were included in this study. We chose to exclusively include female participants in our study, reflecting the fact that approximately 75% of our patient population consisted of women. This decision was made because comparing handgrip strength (HGS) between men and women would not yield meaningful results, given the substantial disparity in strength levels between the sexes.

Patients were diagnosed and recruited at the outpatient department for immunodeficiencies at the Institute of Medical Immunology at the Charité-Universitätsmedizin Berlin between April 2021 and December 2022. Diagnosis of ME/CFS was based on the Canadian Consensus Criteria (CCC) [15] and a minimum of 14 h of PEM [16]. According to the CCC, patients with PCS suffering from persistent fatigue after COVID-19 for at least 6 months were divided into a group with ME/CFS *(n* = 78) and non-ME/CFS (*n* = 66). Patients were excluded from this study in case of relevant comorbidities [9], pre-existing fatigue, or evidence of organ dysfunction. None of the included patients were engaged in strength training or rigorous sports. All patients had to provide proof of previous SARS-CoV-2 infection by positive PCR, antigen test, or serology (SARS-CoV-2 nucleocapsid protein antibodies).

All patients signed informed consent before study inclusion. This study was part of the Pa-COVID-19 study of the Charité-Universitätsmedizin Berlin and was approved by the Ethics Committee of the Charité—Universitätsmedizin Berlin in accordance with the 1964 Declaration of Helsinki and its later amendments (EA2/066/20 and EA2/067/20).

### 2.2. Hand Grip Strength Measurement

HGS of the dominant hand was measured using a digital hand dynamometer (EH101, Deyard, Shenzhen, China) in two separate sessions. Rest time between sessions was 60 min, in which no strenuous physical activity took place. Patients sat in an upright position facing a standard table during measurements of HGS. The forearm of the dominant hand was placed on the table in full supination holding the dynamometer. Under supervision and verbal motivation, the handle was pulled 10 times with maximum force for three seconds, followed by a five-second relaxation phase. Before starting the measurement, the participants were shown two separate demonstrations of how the dynamometer should be used. The dynamometer displayed the highest value reached within these three seconds (measurement in kg), and this single value was then recorded. The attempt with the highest reading out of ten repetitions was recorded as the maximum strength (Fmax), and the average force (Fmean) of each session was calculated. Fatigue ratio (Fmax/Fmean), a measure of the decrease in force during one session, and recovery ratio (Fmean 2/Fmean 1), a measure of recovery of force between both sessions, were calculated.

### 2.3. Questionnaires for Symptom Scoring

Patients’ health-related quality of life was assessed using the 36-Item Short-Form Survey (SF-36), with scores ranging from 0 to 100, with 100 indicating no restriction [17]. Additionally, disease-related disability was scored according to the Bell score, rating the restriction in daily functioning on a scale from 0 to 100, with 100 indicating no restriction [18]. The severity of perceived fatigue was assessed using the Chalder Fatigue Scale (CFQ), ranging from 0 to 33, with 33 indicating maximum fatigue severity [19]. Postexertional malaise (PEM) was evaluated using the DePaul Symptom Questionnaire (PEM-DSQ), ranging from 0 to 46, with 46 indicating maximum PEM severity [16]. Further cardinal symptoms of both PCS and ME/CFS, including fatigue, muscle pain, immunological symptoms, and cognitive impairment, were scored on a scale from 1 to 10, with 10 indicating maximum symptom severity [20]. The fatigue score was calculated as the mean of fatigue, malaise after exertion, need for rest, and daily functioning, and the cognitive score was calculated as a mean of memory disturbance, concentration ability, and mental tiredness based on symptoms recorded with the CCC. The immune score was calculated as a mean of lymph node pain, throat pain, and flu-like feeling. Autonomic dysfunction was evaluated according to the Composite Autonomic Symptom Score (COMPASS 31), ranging from 0 to 100, with 100 indicating maximum autonomic dysfunction [21].

### 2.4. Statistical Analysis

Study data were collected and managed using the REDCap electronic data capture tools hosted at the Charité Universitätsmedizin Berlin [22,23].

Statistical data analyses were performed using IBM SPSS Statistics 29.0 (New York, NY, USA), R 4.3.1 (R Foundation for Statistical Computing, Vienna, Austria, http://www.R-project.org, accessed on 1 August 2023), and GraphPad Prism version 9 for Windows (GraphPad Software, San Diego, CA, USA, www.graphpad.com). All data are presented as the median and interquartile range, or frequency (*n*), and percentage, where appropriate. Comparisons of quantitative parameters between two groups were performed using the nonparametric Mann–Whitney *U* test. Correlation analysis was performed using the nonparametric Spearman coefficient. *p*-values < 0.05 were considered to provide evidence of a statistically significant result.

## 3. Results

### 3.1. Patient Characteristics

This analysis included a cohort of 144 female participants diagnosed with PCS. Among these patients, 78 fulfilled the diagnostic criteria for ME/CFS, while the remaining 66 participants did not. The demographic and clinical characteristics of this study’s population are presented in detail in Table 1.

### 3.2. Comparison between Patient Groups

As illustrated in Table 1, patients with PCS exhibited fatigue, exertional intolerance, and a diverse array of symptoms within both patient groups. The symptoms encompassed PEM, headaches, arthralgia, myalgia, cognitive dysfunction, and autonomic dysfunction, and various others.

**Figure 1:**
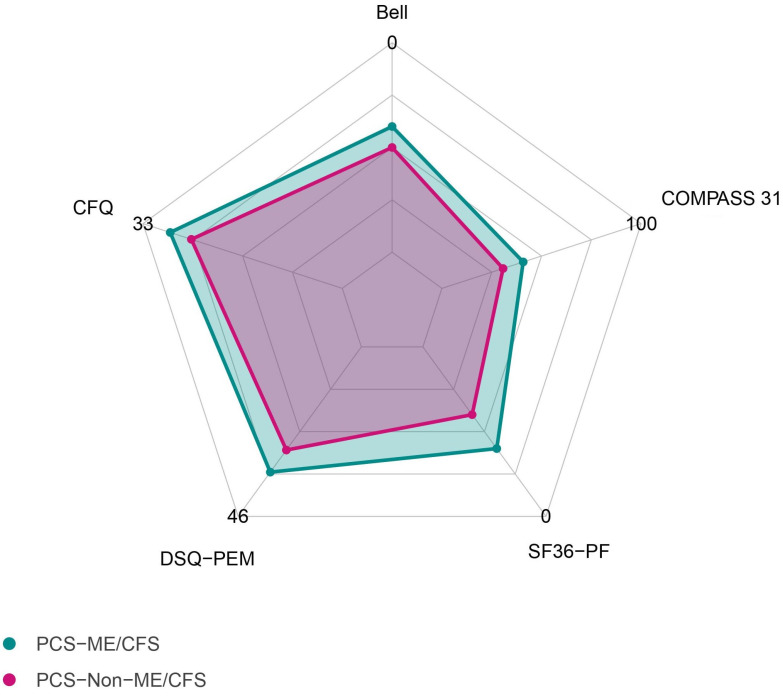
Median scores of questionnaires in patients diagnosed with either PCS or ME/CFS. Bell, disease-related disability according to the Bell score; CFQ, Chalder Fatigue Questionnaire; COMPASS 31, Composite Autonomic Symptom Score; DSQ-PEM, De Paul Symptom Questionnaire for Postexertional Malaise; SF-36-PF, Short-Form 36 Health Survey Physical Function.

As depicted in Table 1 and Figure 1, patients diagnosed with ME/CFS exhibited significantly lower physical function in the SF-36 (*p* < 0.001) and had a significantly higher degree of disability, attributed to their illness as assessed via the Bell score (*p* < 0.001) in comparison to PCS without ME/CFS. Moreover, individuals with ME/CFS exhibited more severe orthostatic intolerance (*p* = 0.006), vasomotor symptoms (*p* = 0.003) fatigue (*p* < 0.001), and PEM (*p* < 0.001). The immune score, calculated as the mean of the three numeric rating scales for the symptoms “painful lymph nodes”, “throat pain”, and “flu-like feeling”, was significantly higher in the ME/CFS group (*p* = 0.006).

### 3.3. Hand Grip Strength and Correlation with Disability and Symptom Severity

Adjusted for age and sex, the median maximum HGS (Fmax 1) in patients with PCS across both groups was below the normal values for the general population provided by the dynamometer manufacturer. Furthermore, we calculated the mean HGS for the initial set of 10 measurements (Fmean 1) and the set taken after a one-hour interval (Fmean 2) and the fatigue ratio (Fmax/Fmean) as a parameter of fatigability within the set of 10 measurements. The recovery ratio was calculated as (Fmean 2/Fmean 1), with values below one indicating impaired recovery after the one-hour interval.

As shown in Table 1, there were little differences in the HGS parameters observed between the two patient cohorts, with only fatigue ratio 2 being higher in patients with ME/CFS. The higher fatigue ratio suggested a more pronounced decline in force during the second session, emphasizing a heightened fatigue response in the ME/CFS patient group.

**Figure 2:**
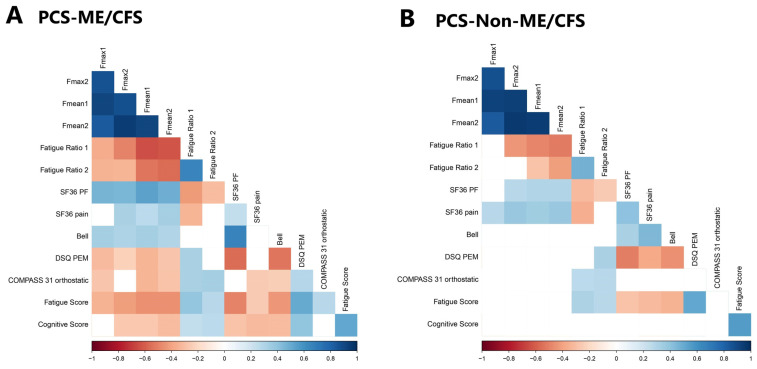
Correlation matrix showing correlation coefficients of parameters of symptom severity and hand grip strength (HGS) for both patient groups. Rectangles indicate the HGS parameters. Nonsignificant coefficients are left blank. (**A**) Patients diagnosed with both PCS and ME/CFS; (**B**) patients diagnosed with PCS but not ME/CFS.

As illustrated in Figure 2 and Table S1, the parameters of HGS demonstrated significant correlations with functional disability and severity of various symptoms between the patient cohorts.

However, distinct correlations of HGS with symptoms were observed when comparing the patient groups. In particular, more and stronger correlations were seen within the ME/CFS group.

Notably, there were strong associations between impaired HGS and higher disability in ME/CFS patients, with a strong positive correlation with the SF-36 physical function along and the Bell score. Furthermore, a lower HGS correlated with more PEM, more pain, fatigue, cognitive impairment, and orthostatic intolerance. In line with this, higher fatigue ratios correlated with more PEM, fatigue, cognitive and orthostatic symptoms.

It is important to note that in patients with PCS but without ME/CFS, a similar correlation of the HGS was only found with the SF-36 physical function and SF-36 pain, but not with PEM, fatigue, cognition and orthostatic intolerance, or with the Bell score. Here, only higher fatigue ratios correlated with more PEM, fatigue, and orthostatic symptoms, while no correlation at all was seen with cognitive impairment.

Furthermore, while the severity of fatigue, pain, and PEM correlated with functional disability, as assessed using the SF36 physical function, in both patient cohorts, a correlation between SF36 physical function and cognition was observed exclusively in the ME/CFS cohort. Furthermore, correlations of more impaired cognition with more pain and PEM and a lower Bell score were found only in the ME/CFS group but not in patients classified as non-ME/CFS, even though the severity of cognitive impairment was similar between the patient groups.

## 4. Discussion

Our study aimed to achieve a thorough comprehension of the symptom patterns evident in both PCS and ME/CFS. Through the utilization of HGS as an objective metric for muscle fatigue, we sought to establish connections between muscle fatigue and functional impairment, as well as symptom severity in patient cohorts of PCS and ME/CFS.

In this study, we observed a diverse range of symptoms among our PCS cohort, in line with the findings of previous studies [8]. Comparing patients with PCS with and without the diagnosis of ME/CFS, those with ME/CFS had various higher-severity symptoms, including fatigue, PEM, orthostatic intolerance, vasomotor, and immune symptoms. Furthermore, patients with ME/CFS experienced a substantially greater degree of disability due to their illness and demonstrated lower levels of physical functioning overall. This observation underscores that although the range of symptom manifestation might be similar between these conditions, post-COVID-ME/CFS is a more severe form of PCS. The diagnosis of ME/CFS following COVID-19 infection is not only linked to an increased symptom burden and an elevated risk of disability, but it also brings about a heightened risk of symptom persistence, as shown in our prior study from 2023 [8].

Currently, the diagnosis of ME/CFS depends on clinical criteria assessed using questionnaires; thus, objective markers would be desirable. Jäkel et al. previously showed HGS to be a sensitive diagnostic test for assessing muscular fatigue and fatigability as well as disease severity in patients with ME/CFS [12]. Furthermore, a lower HGS was associated with a lower Bell score, a more severe PEM, and myalgia in prepandemic postinfectious ME/CFS in our previous study [12]. In a 2023 study by Legler et al., HGS was assessed in patients with PCS, showing that impaired HGS six months after disease onset was a risk factor for symptom persistence [8].

We found that HGS was similarly impaired in patients with PCS, irrespective of ME/CFS diagnosis. However, while SF36 physical function and pain correlated with a diminished HGS in all patients, correlations of impaired HGS and the severity of PEM, cognition, fatigue, and orthostatic intolerance were only found within the patient group with ME/CFS. Most striking is the absence of a correlation between HGS and cognitive impairment in the non-ME/CFS PCS subtype, despite similar symptom severity. Furthermore, cognition correlated with functional disability, pain, and PEM only in patients with ME/CFS but not in those classified as non-ME/CFS.

This points to a common mechanism of muscle fatigue, general fatigue, PEM, cognitive impairment, and orthostatic intolerance in ME/CFS that is distinct from non-ME/CFS PCS. Several studies have elucidated the mechanisms of muscle fatigue, orthostatic intolerance, and cognitive impairment. Central findings in both ME/CFS and PCS str cardiovascular dysfunction and hypoperfusion, as shown by impaired cerebral blood flow and systemic oxygen extraction [24]. The cause for impaired perfusion in ME/CFS and PCS is complex and can be related to both “impaired cardiac preload” and “microcirculatory dysfunction”. The potential mechanisms for these include dysautonomia, endothelial dysfunction, dysfunctional red blood cells, and possibly microclots. Inflammation and mitochondrial impairment can aggravate muscle fatigue. In line with this, histological studies of muscles in PCS revealed capillary injury and rarefication, mitochondrial changes, and inflammation [25,26]. An MRI study revealed higher sodium content in the muscles of patients with ME/CFS compared to those of healthy controls and a correlation with HGS, suggesting the impaired function of ion transport presumably due to hypoperfusion [27].

Our findings in this study suggest that despite the presence of common symptoms across the group of patients with PCS, distinct or additional mechanisms may play a role in ME/CFS. We provided the first evidence for this notion from biomarker studies. In a previous study, we found that HGS was associated with distinct biomarker profiles, suggesting an association with inflammation in PCS and perfusion and muscle damage in ME/CFS [8]. Our observation is in line with that of a very recent study comparing the function and morphology of mitochondria in the muscles of patients with PCS and ME/CFS. Patients with either ME/CFS or PCS exhibited fatigue and impaired mitochondrial function, distinct morphological changes that may indicate mitochondrial damage, were found in ME/CFS only [28]. We also observed differences in endothelial biomarkers and identified a proangiogenic pattern in PCS, whereas no such pattern was observed in ME/CFS [29].

In a study analyzing autoantibodies to G-protein couple receptors (GPCRs), we found an association between fatigue and impaired peripheral perfusion with autoantibodies to vasoregulatory receptors in ME/CFS but not PCS [10]. These findings suggest a potential involvement of autoantibodies in causing or exacerbating hypoperfusion in ME/CFS but not in PCS. Further evidence of the potential role of these autoantibodies comes from studies conducted in patients with ME/CFS after infection. These studies revealed correlations of fatigue, cognitive impairment, and structural central nervous system alterations, with levels of autoantibodies against adrenergic receptors and other GPCR [30,31,32]. Autoantibodies impairing vasoregulation could also be a mechanism explaining the association of muscle fatigue with the cognitive impairment and orthostatic intolerance in ME/CFS observed in the present study.

Importantly, our present study also provides evidence that HGS can serve as an objective marker of physical function in patients with PCS. Additionally, it proves valuable in assessing the severity of key symptoms within the ME/CFS subgroup. Therefore, repeated HGS emerges as an important diagnostic and prognostic tool, enabling an objective evaluation of disability.

Notably, our study is subject to certain limitations. Patients with PCS not fulfilling the ME/CFS criteria constitute a not-well-defined group, characterized by moderate-to-severe fatigue, exertion intolerance, and cognitive impairment. Further limitations include the lack of control group, particularly those who had recovered from COVID-19 or with other chronic diseases. Patients with depression could serve as an interesting control group, given that studies have demonstrated a lower HGS in this population compared to healthy controls. This reduced HGS was linked to the severity of depression and an increased risk of cognitive decline [33]. Furthermore, it is essential to note that this study exclusively involved women patients, as the majority of patients with ME/CFS re women. A recent 2024 study provided evidence of differences in immune and metabolic dysregulation between men and women [34].

## 5. Conclusions

Patients with PCS can present with a broad spectrum of symptoms, encompassing fatigue, PEM, cognitive impairment, pain, and autonomic dysfunction. Notably, HGS impairment was evident in all patients with PCS, with little difference observed in the ME/CFS subgroup. HGS serves as an important measure of muscle fatigue and correlates significantly with both disability and symptom severity in ME/CFS, providing an objective parameter for assessing disease severity.

The observed associations between symptoms and HGS impairment, predominantly identified in the ME/CFS subgroup, suggest the presence of a distinct mechanism or mechanistic link specific to this subset of patients.

This underscores the importance of subgrouping patients with PCS according to ME/CFS criteria in biomarker studies, as potential differences in underlying pathomechanisms could significantly impact the interpretation and applicability of study findings. Understanding these distinctions is essential for advancing our comprehension of post-COVID conditions and tailoring effective diagnostic and therapeutic approaches.

## Figures and Tables

**Table 1:** Demographic and clinical characteristics of this study’s population.

	PCS-ME/CFS (*n* = 78, Median with Range)	PCS-Non-ME/CFS (*n* = 66, Median with Range)	ME/CFS vs. Non-ME/CFS
Age	40 (20–61)	44 (18–67)	*p* = 0.585
BMI	23.71 (14.88–37.97)	24.13 (17.65–41.64)	*p* = 0.675
Time since COVID-19 (months)	10 (6–25)	10.5 (6–23)	*p* = 0.847
Bell	40 (20–90)	50 (20–80)	*p* < 0.001 ***
CFQ	28.5 (13–33)	25 (0–33)	*p* < 0.001 ***
PEM-DSQ	34 (12–46)	28 (0–44)	*p* < 0.001 ***
SF-36			
Physical Functioning	40 (0–95)	60 (0–100)	*p* < 0.001 ***
Role Limitations	0 (0–100)	0 (0–100)	*p* = 0.107
Energy	15 (0–80)	20 (0–60)	*p* < 0.001 ***
Pain	32.5 (0–100)	38.75 (0–100)	*p* = 0.064
Symptom Severity			
Muscle Pain	6 (1–10)	6 (1–10)	*p* = 0.324
Headache	7 (1–10)	6 (1–10)	*p* = 0.115
Joint Pain	5 (1–10)	6 (1–10)	*p* = 0.647
Fatigue Score	8 (1–10)	7 (1–10)	*p* < 0.001 ***
Cognitive Score	6.67 (1.33–10)	6.5 (1–10)	*p* = 0.196
Immune Score	3.33 (1–8.67)	2.33 (1–9)	*p* = 0.006 **
COMPASS 31	40.80(0–76.23)	30.64 (0–62.22)	*p* = 0.002 **
Orthostatic Intolerance	22 (0–40)	16 (0–36)	*p* = 0.006 **
Vasomotor	0 (0–5)	0 (0–3.33)	*p* = 0.003 **
Secretomotor	4.29 (0–12.86)	4.29 (0–12.86)	*p* = 0.485
Gastrointestinal	6.25 (0–15.18)	6.25 (0–17.86)	*p* = 0.187
Bladder	1.12 (0–7.78)	1.11 (0–7.78)	*p* = 0.309
Pupillomotor	1.67 (0–4)	1.67 (1–5)	*p* = 0.634
Hand Grip Strength (in kg)			
Fmax 1	20.1 (5.7–43.6)	19.2 (4.7–33.8)	*p* = 0.884
Fmax 2	18.25 (1.9–41.2)	17.1 (3.10–34.6)	*p* = 0.477
Fmean 1	16.6 (3.19–38.99)	16.07 (4.14–31.85)	*p* = 0.938
Fmean 2	15.07 (1.37–39.05)	14.51 (2.32–30.88)	*p* = 0.990
Fatigue Ratio 1	1.18 (1.01–3.23)	1.17 (1.03–2.11)	*p* = 0.642
Fatigue Ratio 2	1.2 (1.04–2.24)	1.17 (1.03–2.0)	*p* = 0.035 *
Recovery Ratio	0.95 (0.26–1.67)	0.95 (0.47–1.24)	*p* = 0.674

Asterisks mark significant differences between groups (Mann–Whitney test, * *p* < 0.05, ** *p* < 0.01, *** *p* < 0.001). BMI, body mass index; CFQ; Chalder Fatigue Scale; COMPASS 31; Composite Autonomic Symptom Score; fatigue ratio 1/2, ratio of Fmax/Fmean of the first/second session; Fmax 1/2, maximum hand grip strength of the first/second session; Fmean 1/2, mean hand grip strength of the first/second session; ME/CFS, myalgic encephalomyelitis/chronic fatigue syndrome; PCS, post-COVID syndrome; PEM-DSQ; De Paul Symptom Questionnaire for Postexertional Malaise; recovery ratio, ratio of Fmean2/Fmean1, SF-36, Short-Form 36 Health Survey.

## Data Availability

The data presented in this study are available on request from the corresponding author.

## Supplementary Materials

The following supporting information can be downloaded at: https://www.mdpi.com/article/10.3390/jcm13072153/s1, Table S1: Correlations of HGS parameters and clinical parameters.
