# Supplementary material for: Impaired Hand Grip Strength Correlates with Greater Disability and Symptom Severity in Post-COVID Myalgic Encephalomyelitis/Chronic Fatigue Syndrome
Source: J Clin Med. 2024 Apr 8;13(7):2153. doi: 10.3390/jcm13072153 (PMC11012649; doi:10.3390/jcm13072153)
Supplement: Supplementary file 1 [file jcm-13-02153-s001.zip › jcm-2873754-supplementary.pdf]

Table S1. Correlations of HGS parameters and clinical parameters.

	Group	Fmax 1	Fmax 2	Fmean 1	Fmean 2	Fatigue Ratio 1	Fatigue Ratio 2	Recovery Ratio
Bell	PCS-ME/CFS	*r* = 0.34**	*r* = 0.31**	*r* = 0.33**	*r* = 0.29*	*r* = -0.2	*r* = -0.14	*r* = 0.14
	PCS-Non-ME/CFS	*r* = 0.19	*r* = 0.17	*r* = 0.17	*r* = 0.12	*r* = -0.13	*r* = 0.01	*r* = -0.08
CFQ	PCS-ME/CFS	*r* = -0.23*	*r* = -0.19	*r* = -0.26*	*r* = -0.2	*r* = 0.16	*r* = 0.14	*r* = 0.04
	PCS-Non-ME/CFS	*r* = -0.2	*r* = -0.18	*r* = -0.21	*r* = -0.2	*r* = 0.23	*r* = 0.19	*r* = -0.08
DSQ-PEM	PCS-ME/CFS	*r* = -0.32**	*r* = -0.24*	*r* = -0.33**	*r* = -0.27*	*r* = 0.32**	*r* = 0.23	*r* = -0.02
	PCS-Non-ME/CFS	*r* = -0.23	*r* = -0.23	*r* = -0.27	*r* = -0.27	*r* = 0.24	*r* = 0.32*	*r* = -0.07
SF-36								
Physical Function	PCS-ME/CFS	*r* = 0.47***	*r* = 0.45***	*r* = 0.53***	*r* = 0.48***	*r* = -0.43***	*r* = -0.31**	*r* = 0.16
	PCS-Non-ME/CFS	*r* = 0.24	*r* = 0.27*	*r* = 0.3*	*r* = 0.3*	*r* = -0.31*	*r* = -0.26*	*r* = 0.08
Role Limitations	PCS-ME/CFS	*r* = 0.16	*r* = 0.2	*r* = 0.21	*r* = 0.22	*r* = -0.26*	*r* = -0.20	*r* = 0.12
	PCS-Non-ME/CFS	*r* = 0.29*	*r* = 0.3*	*r* = 0.35**	*r* = 0.33**	*r* = -0.3*	*r* = -0.29*	*r* = 0.07
Energy	PCS-ME/CFS	*r* = 0.3**	*r* = 0.38***	*r* = 0.34**	*r* = 0.39***	*r* = -0.32**	*r* = -0.13	*r* = 0.13
	PCS-Non-ME/CFS	*r* = 0.14	*r* = 0.12	*r* = 0.19	*r* = 0.15	*r* = -0.26*	*r* = -0.24*	*r* = -0.007
Pain	PCS-ME/CFS	*r* = 0.19	*r* = 0.32**	*r* = 0.26*	*r* = 0.33**	*r* = -0.33**	*r* = -0.22	*r* = 0.24*
	PCS-Non-ME/CFS	*r* = 0.28*	*r* = 0.38**	*r* = 0.34**	*r* = 0.37**	*r* = -0.35**	*r* = -0.17	*r* = 0.1
COMPASS 31	PCS-ME/CFS	*r* = -0.21	*r* = -0.15	*r* = -0.26*	*r* = -0.22	*r*= 0.3*	*r* = 0.3*	*r* = -0.03
	PCS-Non-ME/CFS	*r* = -0.02	*r* = 0.05	*r* = -0.03	*r* = -0.04	*r* = 0.32**	*r* = 0.22*	*r* = -0.13
Orthostatic	PCS-ME/CFS	*r* = -0.27*	*r* = -0.2	*r* = -0.34**	*r* = -0.29*	*r* = 0.33**	*r* = 0.34**	*r* = -0.11
	PCS-Non-ME/CFS	*r* = -0.01	*r* = 0.03	*r* = -0.06	*r* = -0.07	*r* = 0.27*	*r* = 0.27*	*r* = -0.14
Vasomotor	PCS-ME/CFS	*r* = 0.03	*r* = 0.02	*r* = -0.06	*r* = -0.03	*r* = 0.15	*r* = 0.15	*r* = 0.04
	PCS-Non-ME/CFS	*r* = 0.03	*r* = 0.03	*r* = 0.04	*r* = 0.01	*r* = -0.01	*r* = 0.01	*r* = -0.13
Secretomotor	PCS-ME/CFS	*r* = 0.29*	*r* = 0.28*	*r* = 0.21	*r* = 0.25*	*r* = -0.12	*r* = -0.13	*r* = 0.13
	PCS-Non-ME/CFS	*r* = -0.15	*r* = -0.1	*r* = -0.14	*r* = -0.1	*r* = 0.04	*r* = 0.05	*r* = 0.11
Gastrointestinal	PCS-ME/CFS	*r* = 0.19	*r* = 0.16	*r* = 0.16	*r* = 0.19	*r* = -0.12	*r* = -0.1	*r* = 0.06
	PCS-Non-ME/CFS	*r* = 0.15	*r* = 0.15	*r* = 0.14	*r* = 0.16	*r* = 0.03	*r* = -0.03	*r* = 0.22
Bladder	PCS-ME/CFS	*r* = -0.15	*r* = -0.09	*r* = -0.1	*r* = -0.1	*r* = -0.08	*r* = 0.13	*r* = -0.04
	PCS-Non-ME/CFS	*r* = -0.04	*r* = 0.01	*r* = -0.04	*r* = -0.02	*r* = 0.1	*r* = 0.17	*r* = 0.16
Pupillomotor	PCS-ME/CFS	*r* = 0.26*	*r* = 0.35**	*r* = 0.27*	*r* = 0.32**	*r* = -0.28*	*r* = 0.9	*r* = 0.16
	PCS-Non-ME/CFS	*r* = 0.04	*r* = 0.01	*r* = 0.08	*r* = 0.04	*r* = -0.08	*r* = -0.02	*r* = -0.08
Symptom Severity								
Muscle pain	PCS-ME/CFS	*r* = -0.2	*r* = -0.22	*r* = -0.26*	*r* = -0.27*	*r* = 0.24*	*r* = 0.3 **	*r* = -0.05
	PCS-Non-ME/CFS	*r* = -0.1	*r* = -0.16	*r* = -0.17	*r* = -0.19	*r* = 0.21	*r* = 0.23	*r* = -0.02
Headache	PCS-ME/CFS	*r* = -0.06	*r* = -0.12	*r* = -0.06	*r* = -0.1	*r* = 0.05	*r* = 0.09	*r* = -0.02
	PCS-Non-ME/CFS	*r* = -0.04	*r* = -0.1	*r* = -0.11	*r* = -0.06	*r* = 0.32**	*r* = -0.07	*r* = 0.1
Joint pain	PCS-ME/CFS	*r* = -0.33**	*r* = -0.34**	*r* = -0.43***	*r* = -0.41***	*r* = 0.43***	*r* = 0.36**	*r* = -0.09
	PCS-Non-ME/CFS	*r* = -0.009	*r* = -0.17	*r* = -0.13	*r* = -0.19	*r* = 0.32*	*r* = 0.24	*r* = -0.07
Fatigue Score	PCS-ME/CFS	*r* = -0.35**	*r* = -0.42**	*r* = -0.46**	*r* = -0.46**	*r* = 0.39**	*r* = 0.28*	*r* = -0.07
	PCS-Non-ME/CFS	*r* = -0.044	*r* = -0.06	*r* = -0.11	*r* = -0.11	*r* = 0.32*	*r* = 0.27*	*r* = -0.02
Cognitive Score	PCS-ME/CFS	*r* = -0.22	*r* = -0.26*	*r* = -0.27*	*r* = -0.3**	*r* = 0.24*	*r* = 0.28*	*r* = -0.17
	PCS-Non-ME/CFS	*r* = -0.2	*r* = -0.15	*r* = -0.16	*r* = -0.12	*r* = 0.09	*r* = 0.05	*r* = 0.03
Immune Score	PCS-ME/CFS	*r* = -0.16	*r* = -0.26*	*r* = -0.24*	*r* = -0.31**	*r* = 0.24*	*r* = 0.34**	*r* = -0.17
	PCS-Non-ME/CFS	*r* = -0.008	*r* = -0.04	*r*=- 0.03	*r* = -0.09	*r* = 0.3*	*r* = 0.25	*r* = -0.14

Asterisks mark significant correlations (Spearman Correlation, * $p < 0.05$, ** $p < 0.01$, *** $p < 0.001$). CFQ; Chalder Fatigue Scale; COMPASS 31; Composite Autonomic Symptom Score; Fatigue Ratio 1/2, Ratio of Fmax/Fmean of the first/second session; Fmax 1/2, maximum hand grip strength of the first/second session; Fmean 1/2, mean hand grip strength of the first/second session; ME/CFS, Myalgic Encephalomyelitis/Chronic Fatigue Syndrome; PCS, Post-COVID Syndrome; PEM-DSQ; De Paul Symptom Questionnaire for Post Exertional Malaise; Recovery Ratio, Ratio of Fmean2/Fmean1, SF-36, Short-Form 36 Health Survey.
